# Strand breaks and chemical modification of intracellular DNA induced by cold atmospheric pressure plasma irradiation

**DOI:** 10.1371/journal.pone.0232724

**Published:** 2020-05-06

**Authors:** Hirofumi Kurita, Natsuki Haruta, Yoshito Uchihashi, Takahito Seto, Kazunori Takashima

**Affiliations:** Department of Applied Chemistry and Life Science, Toyohashi University of Technology, Toyohashi, Japan; Kwangwoon University, REPUBLIC OF KOREA

## Abstract

DNA damage in the A549 human lung cancer cell line treated with cold plasma irradiation was investigated. We confirmed that cold atmospheric plasma generated reactive oxygen and nitrogen species (RONS) in a liquid, and the intracellular RONS level was increased in plasma-irradiated cells. However, a notable decrease in cell viability was not observed 24 hours after plasma irradiation. Because RONS induce oxidative damage in cells, strand breaks and chemical modification of DNA in the cancer cells were investigated. We found that 8-oxoguanine (8-oxoG) formation as well as DNA strand breaks, which have been thoroughly investigated, were induced by plasma irradiation. In addition, up-regulation of 8-oxoG repair enzyme was observed after plasma irradiation.

## Introduction

Cold atmospheric pressure plasma (CAP) has been intensively studied due to growing interest in biomedical applications. The feasibility of CAP in biological decontamination, cancer therapy, treatment of chronic wounds, surgical hemostasis, dental care, treatment of skin diseases, and cosmetics has been demonstrated [1–4]. CAP contains a variety of charged particles, reactive oxygen and nitrogen species (RONS), light, and electric fields. Biological and medical applications of CAP have been developed using the above properties. For practical use, the biological influence of CAP treatment on living cells and organs therefore needs to be well understood. Among the various applications described above, cancer therapy is one of the most promising targets of plasma medicine [5, 6]. Cell culture medium irradiated with CAP, so-called plasma-activated medium, shows antitumor effects, similar to direct plasma irradiation of tumor cells or tissue. For example, plasma-activated medium selectively kills glioblastoma brain tumor cells [7–9] and ovarian clear-cell carcinoma [10]. Furthermore, CAP treatment of cancer cells is expected to trigger a cancer-specific immune response [11, 12]. The common and central issues in this field are selective induction of apoptosis in cancer cells [13–15], the role of RONS generated during CAP treatment of cancer cells as the trigger of oxidative stress, and the different signaling pathways in cells [16–20]. For example, hydrogen peroxide is considered a key factor for its antitumor effect [21], and synergistic effects of hydrogen peroxide and reactive nitrogen species in the antitumor effects have been demonstrated [9, 22].

Although several mechanisms have been suggested, our understanding of the molecular mechanisms is incomplete. Recent progress in biomedical applications of non-thermal plasmas shows that the biological effects are mainly due to oxidative reactions induced by RONS produced by exposure to the plasma [23, 24]. For example, one proposed molecular mechanism of the antitumor effect is DNA damage-associated cell death. The biological significance of damage to DNA by RONS depends on the extent of damage, where the damage occurs in the genome, and how fast it can be repaired. Damage to DNA usually halts DNA replication and cell division until repair is complete. Several modified DNA bases can mispair [25]. Furthermore, Guo et al. demonstrated DNA-protein crosslinks induced by CAP treatment [26]. Therefore, damage to DNA induced by plasma irradiation can be a genotoxin and can lead to cell death such as p53-mediated apoptosis [27–29].

For the above reasons, DNA is one of the most important biomolecular targets for investigating the effects of exposure to plasma. Oxidative damage is induced in DNA when DNA is exposed to plasma [30]. Over the last decade, many studies have attempted to characterize DNA damage and the associated cellular responses induced by plasma irradiation. In the early stage of the investigation, most reports used isolated plasmid DNA molecules in liquids, and the analysis was based on gel electrophoresis. For example, oxidative damage is induced by exposure to plasma, resulting in single-strand breaks (SSBs) and double-strand breaks (DSBs), which can be separated and visualized with conventional agarose gel electrophoresis [31]. Furthermore, investigations of DNA in plasma-irradiated cells have been reported. For example, the single-cell gel electrophoresis assay, also known as the comet assay, is a versatile method for measuring DNA damage [28, 32]. To study plasma interactions with DNA, the plasma jet readily induces DNA strand breaks in artificial models consisting of tissue fluid, tissue, and cells, surprisingly without any significant rupture of the phospholipid membrane [33, 34]. On the other hand, chemical modification of DNA bases may be induced by plasma irradiation. For example, Okazaki et al. and Attri et al. showed that plasma irradiation generates 8-oxoguanine (8-oxoG) in aqueous DNA [35, 36]. Okazaki et al. also showed that plasma irradiation generates 8-oxoG in *ex vivo* rat liver. 8-oxoG generation and strand breaks in cancer cells induced by plasma irradiation were also demonstrated by Joh et al. [37] and Choi et al. [38]. However, these previous studies did not discuss the specificity of 8-oxoG generation in cellular DNA [35, 37, 38]. Furthermore, these reports did not investigate the correlation between cell viability and the extent of DNA damage. Therefore, the biological significance of damage to DNA including 8-oxoG generation and strand breaks induced by plasma irradiation remain to be investigated.

In this study, A549 cells, which are a human lung cancer cell line, were irradiated with an atmospheric pressure plasma jet (APPJ) generated with helium. The cells were suspended in Dulbecco’s modified phosphate buffered saline without magnesium chloride and calcium chloride (D-PBS (−)), irradiated with the plasma jet, and then DNA damage, i.e., strand breaks and generation of 8-oxoG, was assessed with the comet assay and immunostaining immediately after plasma irradiation. Because generation of 8-oxoG and high cell viability 24 hours after plasma irradiation were observed, up-regulation of an 8-oxoG repair enzyme was also investigated.

## Materials and methods

### Cell line and cell culture

The human lung tumor cell line A549 (RIKEN BRC) was used in this study. Cells were grown in Dulbecco’s modified Eagle’s medium (DMEM) supplemented with 4 mM l-glutamine (Wako Pure Chemicals), 10% fetal bovine serum (FBS, One Shot fetal bovine serum, Thermo Fisher Scientific), and penicillin/streptomycin (PS) (Wako Pure Chemicals) at 37°C in 5% CO_2_. Cells at 50-70% confluence in T25 or T75 flasks were treated with 0.25% trypsin-EDTA (Wako Pure Chemicals), harvested by centrifugation, and suspended in DMEM/10% FBS/PS. The cells were then washed with D-PBS (−) (Wako Pure Chemicals) before final re-suspension, and the cell concentration was adjusted.

### Plasma source and plasma treatment

**Fig 1:**
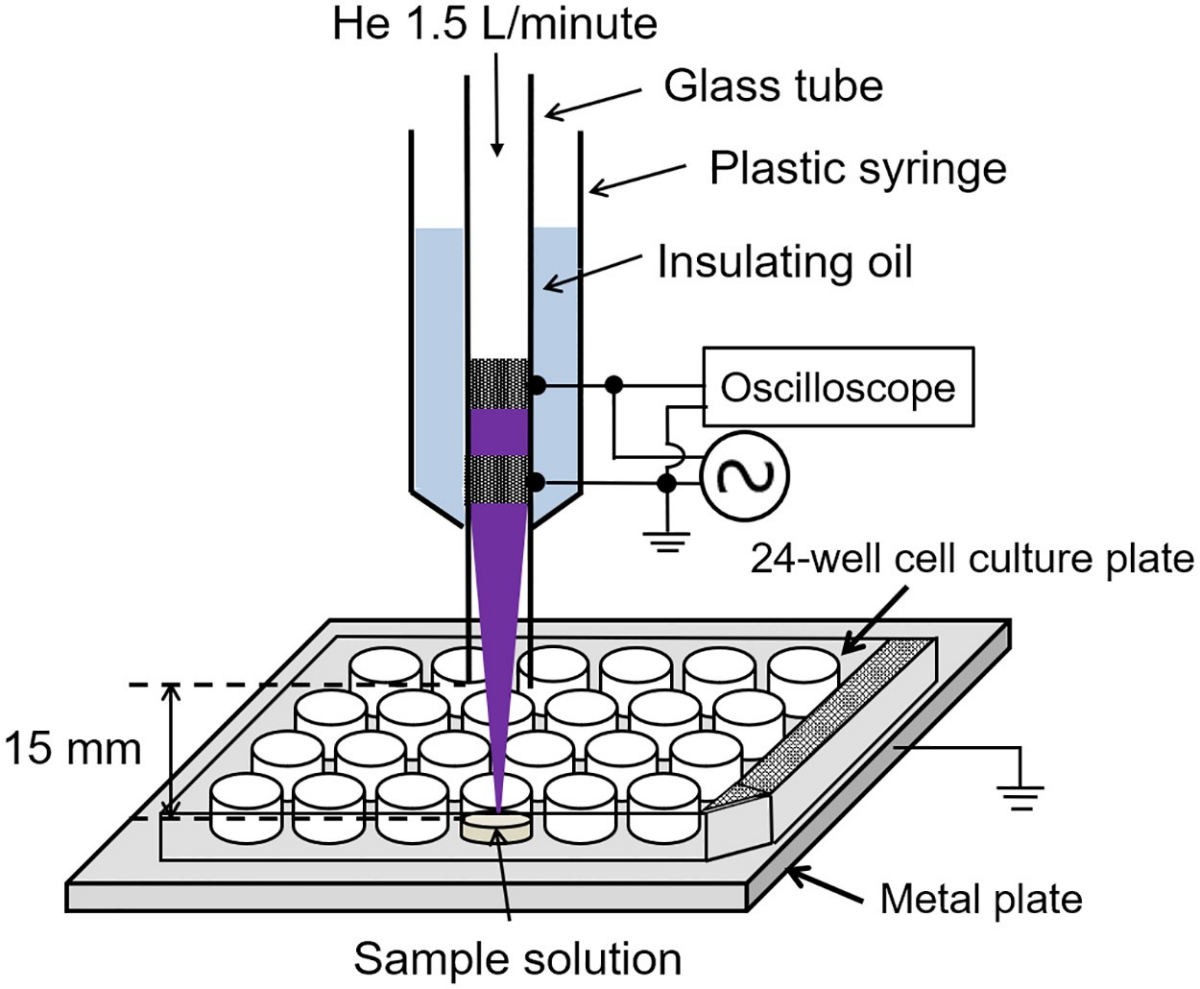
Schematic illustration of the experimental apparatus.

Fig 1 shows a schematic illustration of the experimental apparatus for exposing samples to an APPJ. The APPJ consisted of a quartz glass tube with an inner/outer diameter 2.1/2.6 mm, with two copper tape electrodes (10 mm width) spaced 5 mm apart. One electrode was powered (10 kV_0-p_ sinusoidal voltage at 17 kHz) and the other was grounded. The distance between the grounded electrode and the nozzle was set to 10 mm. The glass tube was fixed in a plastic syringe (Terumo) filled with insulating oil (3M Fluorinert FC-43). Helium was used as a carrier gas at 1.5 L/minute. One milliliter of the sample solution, e.g., cell suspension, was added to one well of the 24-well tissue culture test plate (TPP). The gap between the nozzle and the surface of the sample solution was fixed at 15 mm. All experiments were carried out at room temperature.

### Reactive species produced in liquids

Production of hydroxyl (OH) radicals and superoxide (O_2_^−^) in the plasma-irradiated liquid was confirmed with electron spin resonance (ESR) spin-trapping. One milliliter of 5-(2,2-Dimethyl-1,3-propoxy cyclophosphoryl)-5-methyl-1-pyrroline N-oxide (CYPMPO) (10 mM) (Shidai Systems) in D-PBS (−) was added to one well of a 24-well tissue culture test plate. Following plasma irradiation, the sample was transferred to a flat cell, and the ESR signals were measured using an ESR spectrometer (JES-X310, JEOL Resonance) operating at 9.42 GHz and at 100 kHz field modulation. The settings of the spectrometer were: center magnetic field: 336.0 mT; field width: ±5.0 mT; microwave power: 10 mW; modulation amplitude: 0.2 mT; time constant: 0.3 seconds.

We also estimated the concentration of H_2_O_2_, NO_2_^−^, and NO_3_^−^ in the plasma-irradiated liquid. The detailed experimental procedure was described previously [39]. The H_2_O_2_ concentration was determined with the Amplex Red Hydrogen Peroxide kit (Thermo Fisher Scientific) and a multimode microplate reader (Varioskan FC, Thermo Scientific). The concentration of NO_2_^−^ ([NO_2_^−^]) was determined with the NO_2_/NO_3_ Assay Kit-FX (Fluorometric) (Dojindo). The concentration of NO_3_^−^ ([NO_3_^−^]) was determined with the NO_2_/NO_3_ Assay Kit-C II (Colorimetric) (Dojindo). First, [NO_2_^−^+NO_3_^−^] was estimated with the colorimetric method, and then [NO_3_^−^] was obtained by subtraction of [NO_2_^−^] from [NO_2_^−^+NO_3_^−^]. The concentration was determined from the standard reference curve.

### Analysis of intracellular reactive species

Reactive species in cells were analyzed with a general oxidative stress fluorescence probe 5-(and-6)-chloromethyl-2’,7’-dichlorodihydrofluorescein diacetate, acetyl ester (CM-H_2_DCFDA) (Thermo Fisher Scientific). Before plasma irradiation, A549 cells suspended in D-PBS (−) were mixed with 10 *μ*M CM-H_2_DCFDA for 0.5 hours. One milliliter of the cell suspension in one well of a 24-well tissue culture test plate at the concentration of 2.0 × 10^5^ cell/ml was irradiated with the APPJ. After plasma irradiation of the cell suspension, detection of intracellular reactive species was performed with flow cytometry with a CytoFLEX flow cytometer (Beckman Coulter). At least 10,000 cells were analyzed for each experimental point.

### Cell viability assay

A549 cells were suspended in one well of a 24-well tissue culture test plate at a concentration of 2.0 × 10^5^ cells/ml in 1 ml D-PBS (−) and were irradiated with the APPJ. After plasma irradiation, the cell suspension was centrifuged at 400 × g for 5 min at 4°C, and then the cells were resuspended in DMEM/10%FBS/PS and cultured for 24 hours at 37°C in 5% CO_2_. The cells in medium, any washes, and cells that were detached by trypsinization were harvested by centrifugation. All harvested cells were loaded with the dead-cell staining dye 7-AAD (Beckman Coulter), and analyzed with flow cytometry.

### Comet assay

To assess cellular DNA damage in plasma-irradiated cells, the modified alkaline comet assay was performed with the hOGG1 FLARE Assay Kit (Trevigen). This kit includes human 8-oxoguanine glycosylase (hOGG1), which is a DNA repair enzyme that recognizes and removes 8-oxoG. A549 cells in one well of a 24-well tissue culture test plate at a concentration of 2.0 × 10^5^ cells/ml in 1 ml D-PBS (−) were irradiated with the APPJ. Immediately after APPJ irradiation, the cell suspension was mixed with molten agarose at 37°C at a ratio of 1:10 (v/v), and 40 *μ*l of the mixture was immediately pipetted onto a FLARE slide. Then the slide was placed flat at 4°C in the dark for 10–30 minutes. The slide with the cured gel was immersed in cell lysis solution at 4°C for more than 60 minutes. The slide was then placed in 1× FLARE buffer for 3 × 30 minutes at room temperature. Following gel equilibration, the hOGG1 enzyme solution was pipetted onto the gel, and the slide was placed flat at 37°C in a humid chamber for 30 minutes. After hOGG1 treatment, the slide was washed with 1× FLARE buffer once, and then the slide was immersed in alkaline buffer in the dark at room temperature for 30 minutes. The slide was electrophoresed at 35 V (1 V/cm) in the alkaline buffer for 30 minutes at 4°C. Then, the slide was immersed in deionized water for 2 × 5 minutes and 70% ethanol for 5 minutes, and then dried at room temperature. Finally, DNA was stained with SYBRgold (1:10000 in TE buffer: 10 mM Tris-Cl (pH 7.5), 1 mM EDTA) for 30 minutes before fluorescent images were acquired with a fluorescence microscope (Eclipse TE2000-U, Nikon) equipped with a 40× objective lens. The images were analyzed with OpenComet software.

### Detection of up-regulation of the 8-oxoG repair enzyme in plasma-irradiated cells

To assess the up-regulation of the 8-oxoG repair enzyme, molecular beacons (MBs) were used [40]. The MBs were transfected into cells before APPJ treatment. MBs with 8-oxoG were commercially synthesized by Tsukuba Oligo Service Co., Ltd. Japan. The complementary sequences that form a double helix structure are FAM-5’-GCA CT8 AAG CGC CGC GCT TCA GTG C-3’-BHQ-1 (FAM is a fluorescent dye; BHQ-1 is a quencher; 8 is 8-oxoG). A control experiment that uses MBs without 8-oxoG (commercially synthesized by Fasmac, Japan) was also performed. Prior to use, MBs were incubated at 95°C for 3 minutes, and then left to anneal by cooling slowly (> 3 hours) to room temperature in the dark, allowing formation of the correct conformation of the oligonucleotide. Transfection was performed with the SuperFect Transfection Reagent (Qiagen) according to the manufacturer’s instructions. The transfected cells were harvested and re-suspended in D-PBS (−). The cell suspension was irradiated with the plasma jet using the conditions described above. After plasma irradiation of the cell suspension, the cell suspension was centrifuged at 400 × g for 5 minutes at 4°C, and then the cells were resuspended and cultured for 24 hours at 37°C in 5% CO_2_. Following the 24-hours incubation, the cells were trypsinized and resuspended in D-PBS (−). Finally the cells were analyzed with flow cytometry. At least 10,000 cells were analyzed for each experimental point.

## Results

### Production of RONS in plasma-irradiated liquid and cells

**Fig 2:**
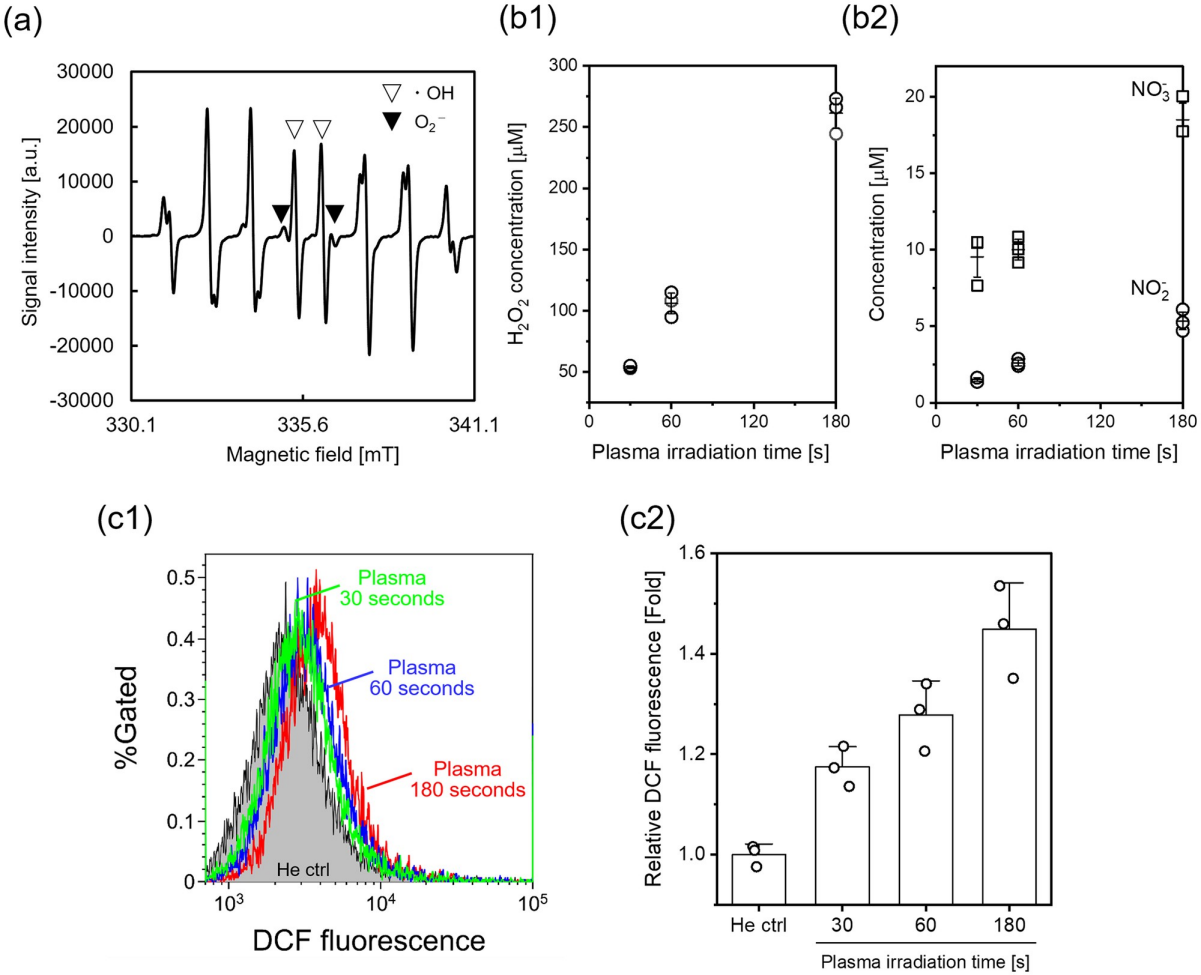
RONS production in the plasma-irradiated liquid and cells. (a) ESR spectrum of the plasma-irradiated CYPMPO solution. The plasma irradiation time was 180 seconds. The spin adduct signals of the OH radical and superoxide can be distinguished by the peaks in the central part; the solid triangle markers indicate the superoxide adduct, and the open triangles indicate the OH adduct. One milliliter of 10 mM CYPMPO in D-PBS (−) was added to one well of a 24-well tissue culture test plate before exposure to the helium plasma jet. (b) Concentration of (b1) H_2_O_2_ and (b2) NO_2_^−^ and NO_3_^−^ in plasma-irradiated D-PBS (−). Data are expressed as the mean±standard deviation (SD) of triplicate measurements. (c) Intracellular RONS production in the plasma-irradiated A549 cells. A549 cells were treated with the He plasma jet for 30, 60 and 180 seconds, and then RONS production was detected with flow cytometry using CM-H_2_DCFDA. (c1) Typical flow cytometry histograms and (c2) median fluorescence intensity normalized to the He ctrl. “He ctrl” indicates a control experiment, in which the cell suspension was exposed to He gas flow for 180 seconds. Data are expressed as the mean±SD of triplicate measurements. Statistical significance was recognized at *p* = 0.0026 for 30 seconds, *p* = 0.00246 for 60 seconds, and *p* = 0.00122 for 180 seconds vs. the He ctrl as determined with the Student’s *t*-test.

Several RONS, e.g., OH radicals, cause DNA damage, which may be measured as strand breakage and/or chemical modification of DNA bases or 2-deoxyribose. First, RONS production in D-PBS (−) during plasma irradiation was investigated using ESR with the spin trapping reagent, CYPMPO, which can detect OH radicals and superoxide. Fig 2(a) shows the ESR spectrum of plasma-irradiated D-PBS (−) with CYPMPO obtained immediately after plasma irradiation for 180 seconds. Because the ESR spectrum consists of overlap of two radical species due to the hyperfine interaction in the spin adducts, all peaks except the four peaks near the center of the spectrum correspond to the CYPMPO-OH spin adduct and CYPMPO-O_2_^-^ spin adduct. The four peaks near the center of the spectrum denoted by open triangle markers are due to the OH adduct, whereas the next two peaks denoted by solid triangles are due to the O_2_^-^ adduct. This result suggests production of OH radicals and superoxide in D-PBS (−) during plasma irradiation.

Fig 2(b) shows the concentration of H_2_O_2_, NO_2_^−^, and NO_3_^−^ after plasma irradiation with different plasma irradiation time. This result indicates that the concentration of long-lived RONS increased with increasing plasma irradiation time, and approximately 250 *μ*M H_2_O_2_, 5 *μ*M NO_2_^−^ and 19 *μ*M NO_3_^−^ were produced during 180 seconds of plasma irradiation. Compared with hydrogen peroxide, the nitrite and nitrate concentrations were low. Therefore, hydrogen peroxide is considered as a major long-lived RONS in the plasma-irradiated liquid in this study.

Then, intracellular RONS production was measured by using the RONS-reactive fluorescent probe CM-H_2_DCFDA. This probe selectively detects RONS, although the probe does not distinguish individual types of RONS well. This probe can penetrate inside the cell, and then a portion of the probe is degraded by cellular esterase. When the probe reacts with several RONS, fluorescence intensity is increased. The probe was loaded into the human lung cancer cell line, A549, suspended in D-PBS (−), and then the cell suspension was irradiated with the plasma jet. The plasma irradiation time was set to 30, 60, and 180 seconds. The plasma-irradiated cell suspension was analyzed with flow cytometry immediately after the plasma irradiation. Fig 2(c) shows the result of the flow cytometric analysis. Plasma irradiation resulted in a significant change in the flow cytometric profile relative to the control (He gas 180 seconds), suggesting that the intracellular RONS level was increased immediately after plasma irradiation.

### Cell viability

**Fig 3:**
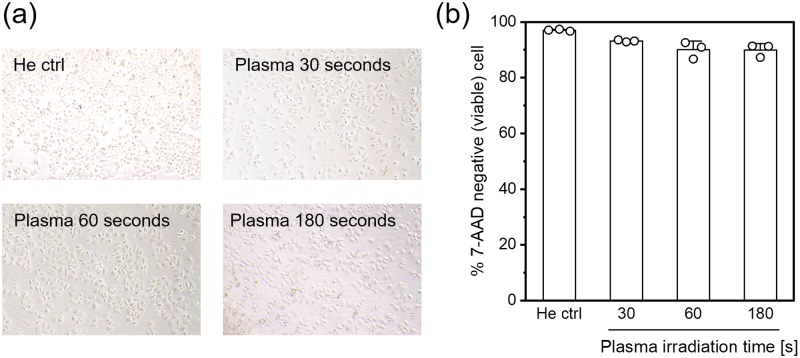
Microscopic observation and cell viability of plasma-irradiated A549 cells. (a) Bright field images of A549 cells 24 hours after He plasma irradiation. “He ctrl” indicates the control experiment in which the cell suspension was exposed to He gas flow for 180 seconds. (b) Cell viability 24 hours after He plasma irradiation as determined with flow cytometry. The cells were stained with the dead-cell staining dye, 7-AAD. Data are expressed as the mean±SD of triplicate measurements. Statistical significance was determined with the Student’s *t*-test, *p* < 0.001 for 30 seconds, *p* = 0.01686 for 60 seconds, and *p* = 0.00642 for 180 seconds vs. He ctrl.

Fig 3(a) shows microscopic images of plasma-irradiated A549 cells acquired 24 hours after plasma irradiation. No significant change in cell growth was observed in the various irradiation conditions. Fig 3(b) shows the cell viability 24 hours after plasma irradiation as determined with flow cytometry. A slight decrease in the cell viability was observed, but more than 89% of the cells were viable in each condition. These results suggest that plasma irradiation and the irradiation condition used in this study did not result in notable cell death.

### Strand breaks in the plasma-irradiated DNA

**Fig 4:**
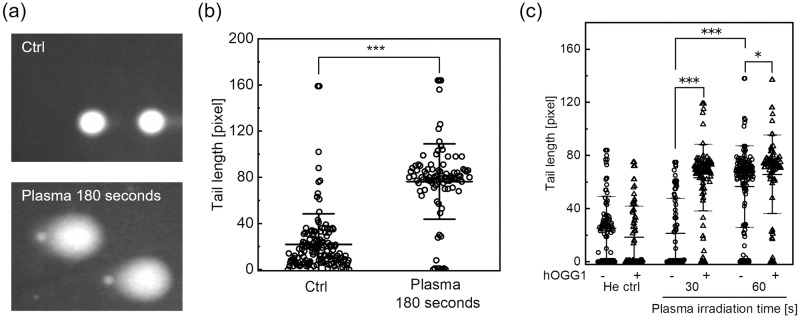
Strand breaks and chemical modification in A549 cells induced by He plasma jet treatment. Cells were treated with the plasma jet, and DNA damage levels were measured using the alkaline comet assay. (a) Typical fluorescence images acquired in the alkaline comet assay without hOGG1 treatment. “Plasma 180 seconds” indicates He plasma jet irradiation for 180 seconds. “Ctrl” indicates the control experiment in which the cell suspension was exposed to He gas flow for 180 seconds at the same flow rate. (b) The tail length was measured as the DNA damage level. Each dot represents a single cell, with a minimum of 90 cells counted for each experimental condition. Data are expressed as mean±SD. Statistical significance was determined with the Student’s *t*-test, ***p < 0.0001. (c) The alkaline comet assay combined with human 8-oxoguanine glycosylase (hOGG1) treatment. The tail length with different plasma irradiation times and in the presence or absence of hOGG1 treatment is shown. Each dot represents a single cell, with a minimum of 80 cells counted for each experimental condition. Data are expressed as the mean±SD. Statistical significance was recognized at *p = 0.02238 and ***p < 0.0001 as determined with the Student’s *t*-test.

Strand breaks were analyzed with the alkaline comet assay without hOGG1 treatment immediately after plasma irradiation. The cells were embedded in low melting agarose following He plasma irradiation of the cell suspension, and then lysed. The DNA was electrophoresed and then stained with fluorescent dyes. Fig 4(a) shows typical fluorescence images acquired with microscopic observation. When exposed to an electrophoretic field, damaged cellular DNA is separated from intact DNA, yielding a “comet tail” shape under the microscopic field. Most comets in the untreated control showed no fluorescent tails, indicating that nuclear DNA was intact. In contrast, plasma irradiation increased the number of typical comets with tails. From the acquired images, the tail length was measured as the DNA damage level, and the results are summarized in Fig 4(b). Plasma irradiation for 180 seconds resulted in a significant increase in the tail length relative to the control. Thus, He plasma jet irradiation can induce strand breaks.

### Chemical modification of the plasma-irradiated DNA

Chemical modification of DNA bases, i.e., production of 8-oxoG was assessed with the alkaline comet assay after cells were treated with hOGG1. hOGG1 is a DNA repair enzyme that recognizes and removes 8-oxoG [25]. Cells embedded in agarose gel were treated with hOGG1, resulting in SSBs due to removal of 8-oxoG, before alkaline electrophoresis. Thus, the tail length increases if DNA has 8-oxoG. Fig 4(c) shows the result of the alkaline comet assay after cells were treated with hOGG1. The plasma irradiation time was set to 30 and 60 seconds. In the control experiment (He gas 60 seconds), hOGG1 treatment did not result in a significant change in the tail length. On the other hand, plasma irradiation for 30 or 60 seconds resulted in a significant increase in the tail length in the presence of hOGG1 treatment. Compared to the control experiment (He gas 60 seconds), no significant increase in the tail length was observed when cells were treated with 30 seconds of plasma irradiation without hOGG1 treatment; therefore, 30 seconds of plasma irradiation did not induce strand breaks in A549 cells. However, He plasma irradiation for 60 seconds resulted in a significant increase in the tail length relative to plasma irradiation for 30 seconds. These results suggest that plasma irradiation that cannot induce strand breaks may induce chemical modification of DNA bases.

To confirm the production of 8-oxoG in A549 cells induced by He plasma jet irradiation, immunofluorescence staining was also performed. Immediately after He plasma jet irradiation, the cells were fixed, treated with RNase and HCl, permeabilized, and stained with an anti-8-oxoG antibody labeled with a fluorescent dye. S1 Fig shows the result of immunostaining detected with flow cytometry. Plasma irradiation for 180 seconds resulted in a change in the flow cytometric profile relative to the untreated control. These results demonstrated that He plasma jet irradiation induces 8-oxoG production in A549 genomic DNA.

### Up-regulation of the 8-oxoG repair enzyme induced by plasma irradiation

**Fig 5:**
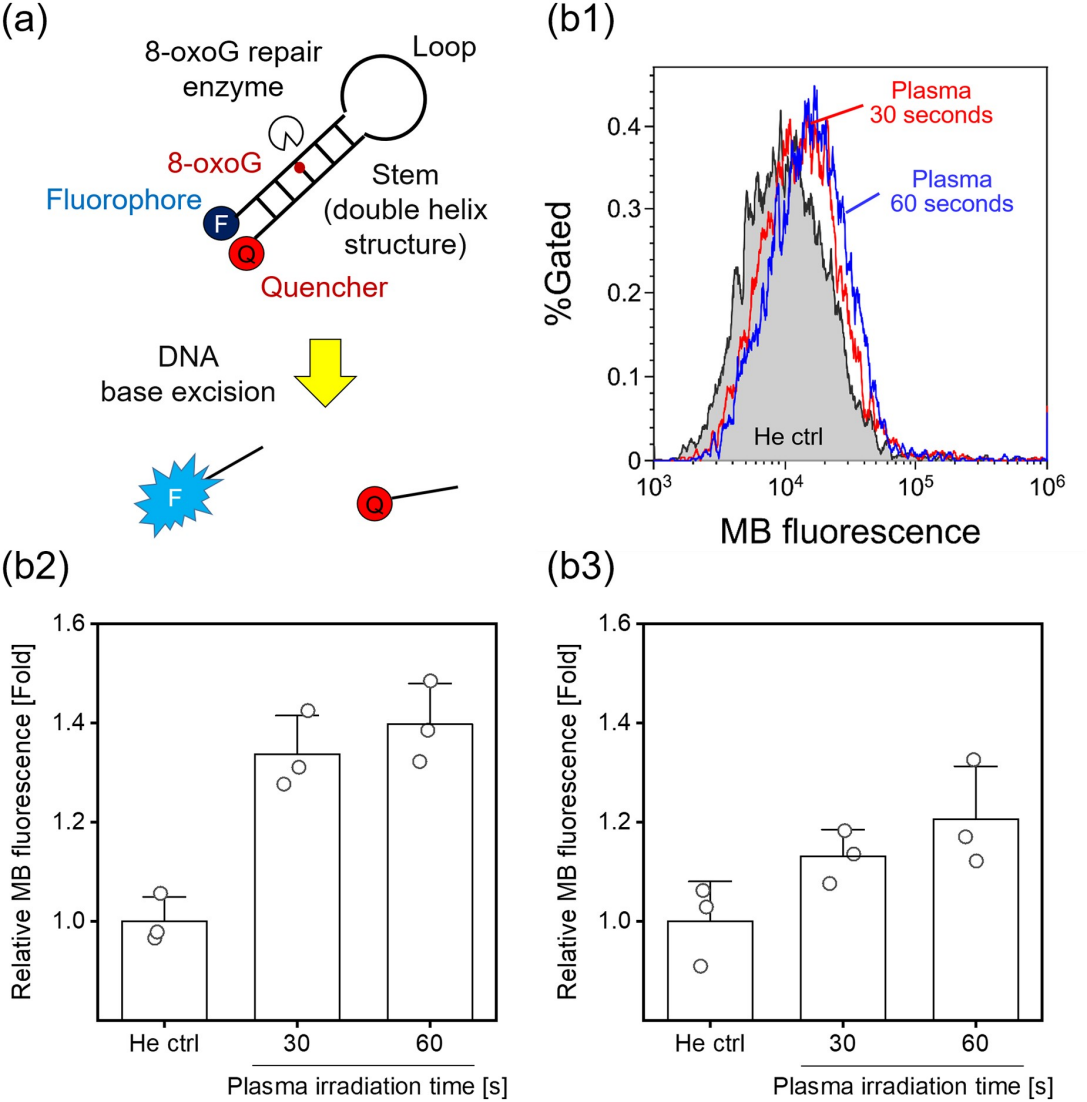
Detection of up-regulation of the DNA repair enzyme after plasma irradiation. (a) A schematic of the experimental principle. A MB containing a fluorescent dye and a quencher at opposite ends and an 8-oxoG:C pair in the stem region. Up-regulation of the 8-oxoG repair enzyme results in a fluorescence increase due to removal of 8-oxoG. (b) Flow cytometric analysis of up-regulation of the 8-oxoG repair enzyme 24 hours after He plasma irradiation. (b1) Typical flow cytometry histograms and (b2) median fluorescence intensity normalized to the He ctrl. “He ctrl” indicates the control experiment in which the cell suspension was irradiated with He gas flow for 60 seconds. Data are expressed as the mean±SD of triplicate measurements. Statistical significance was recognized at *p* = 0.00311 for 30 seconds and *p* = 0.00197 for 60 seconds vs. He ctrl as determined with the Student’s *t*-test. (b3) Control experiment that uses an unmodified MB. Data are expressed as the mean±SD of triplicate measurements. Statistical significance was not found (*p* = 0.07745 for 30 seconds and *p* = 0.05575 for 60 seconds vs. He ctrl) as determined with the Student’s *t*-test.

MBs were used to assess the up-regulation of DNA repair enzymes in live cells after plasma irradiation. Fig 5(a) shows a schematic of the experimental principle [40]. MBs are oligonucleotides that adopt a stem-and-loop structure and carry a 5’-fluorescent moiety and a 3’-nonfluorescent quenching moiety at opposite ends. The MBs used in this study also contain an 8-oxo-G:C base pair in the stem structure. Scission of the MB stem by 8-oxoG repair enzymes leads to separation of the fluorophore-quencher pair, resulting in an increase in fluorescence that directly correlates with the extent of the activity of the DNA repair enzyme. MBs were transfected into A549 cells 1 day before plasma irradiation. The cells were trypsinized and suspended in D-PBS (−), and the cell suspension was irradiated with the He plasma jet. After plasma irradiation, the cells were cultured for 24 hours, and then the cells were analyzed with flow cytometry. Fig 5(b) shows the results of flow cytometric analysis of up-regulation of the 8-oxoG repair enzyme using the MBs. As shown in Fig 5(b1), plasma irradiation resulted in a change in the flow cytometry histograms relative to the control (He gas 60 seconds). Fig 5(b2) shows median fluorescence intensity normalized to the He ctrl. Compared with the He ctrl, He plasma irradiation for 30 or 60 seconds resulted in a significant increase in median fluorescence intensity. However, the control experiment that used an MB without 8-oxoG showed no significant increase in median fluorescence intensity (Fig 5(b3)). These results suggest that He plasma jet irradiation up-regulates the 8-oxoG repair enzyme in A549 cells.

## Discussion

In this paper, strand breaks and chemical modification in plasma-irradiated cells induced by He plasma jet irradiation were investigated. Many previous reports have already demonstrated DNA damage induced by cold plasma irradiation. In these reports, different experimental conditions have been employed including direct and indirect plasma irradiation; in addition, the experimental time points of DNA damage assessment were also different. We performed He plasma jet irradiation of cell suspensions, and DNA damage was assessed immediately after the plasma irradiation. Fig 4 demonstrated that He plasma jet irradiation induced strand breaks. The cells were embedded in gel immediately after the He plasma jet irradiation; therefore, strand breaks must have been induced during the plasma irradiation. Hirst et al. demonstrated that DNA damage, which is uniform across different cell types, was inflicted by a plasma exposure of only 30 seconds without a severe decrease in cell viability [32]. Fig 3 indicates that He plasma jet irradiation did not result in a notable decrease in cell viability; therefore, our results agreed with those of Hirst et al., although the plasma irradiation conditions were different. In both this paper and that of Hirst et al., an alkaline comet assay was used to assess DNA damage. This assay can detect both SSBs and DSBs. Therefore, the DNA damage displayed in Fig 4 (without hOGG1 treatment) contained both SSBs and DSBs. Choi et al. performed not only an alkaline but also a neutral comet assay, which detects only DSBs, and they showed that cold plasma irradiation primarily generates SSBs [38]. Taken together, He plasma jet irradiation induced strand breaks in an irradiation time-dependent manner, and SSBs may have been more frequently generated than DSBs, although a notable reduction in cell viability was not observed.

Chemical modification, i.e., generation of 8-oxoG, was also assessed immediately after He plasma jet irradiation. Fig 4(c) showed that 8-oxoG generation was induced during He plasma jet irradiation. Once again, although previous studies also demonstrated that cold plasma irradiation induces 8-oxoG generation, the specificity of 8-oxoG generation in genomic DNA was not discussed [35, 37, 38]. These assessments were based on immunostaining. In contrast, our alkaline comet assay combined with hOGG1 treatment can specifically detect 8-oxoG in DNA. Furthermore, 8-oxoG detection with immunostaining investigated in this study included RNase treatment. Moreover, activation of DNA repair enzymes was also observed in the plasma-irradiated cells. Therefore, our results strongly suggest that 8-oxoG generation in intracellular DNA was induced by cold plasma irradiation. In addition, the dose dependency of 8-oxoG generation was not well studied in previous studies. Fig 4(c) clearly showed that 8-oxoG generation was induced by 30 seconds of He plasma jet irradiation, which cannot induce strand breaks. From this and the discussion described above, 8-oxoG generation must be a more readily induced type of DNA damage than strand breaks.

Although 8-oxoG generation in the plasma-irradiated cells was confirmed, a notable reduction in cell viability 24 hours after plasma jet irradiation was not observed (Fig 3). Therefore, up-regulation of the DNA repair enzyme was investigated. Fig 5 demonstrated up-regulation of the 8-oxoG repair enzyme in living cells irradiated with the He plasma jet. Rosani et al. reported that He plasma irradiation does not cause morphological changes in human corneal tissue but causes a transient and slight increase in hOGG1 protein expression [41]. Our results agreed with those of Rosani et al.; however, this paper did not investigate 8-oxoG generation. Therefore, a direct correlation between 8-oxoG generation in DNA and up-regulation of its repair enzymes induced by cold plasma irradiation has not been reported before. Most previous studies have reported a correlation between an obvious reduction in cell viability and its mechanism, especially apoptosis induction, after cold plasma irradiation. In contrast, this study showed that although we used weaker plasma irradiation that did not induce notable cell death, intracellular DNA was damaged and repaired following plasma irradiation. Cellular responses induced by such weak plasma irradiation had not been well studied; however, these cellular responses could not be observed by the plasma irradiation protocol that was performed in most previous studies. Therefore, this study proposes a new concept for investigation of cellular responses in plasma medicine.

RONS production in the plasma-irradiated liquid and cells was observed as a dominant factor in DNA damage. Fig 2(a) shows production of OH radicals and superoxide. Tani et al. investigated the formation of OH radicals and superoxide in aqueous solution exposed to a He plasma jet using the same spin-trapping reagent CYPMPO [42]. The obtained ESR spectrum showing production of OH radicals and superoxide agreed with those of Tani et al., although the plasma irradiation conditions were different. In our previous study, we proposed that OH radicals may play an important role in causing DNA damage when DNA is suspended in a liquid [43]. Because the lifetime of OH radicals is very short (less than 1 millisecond), the radicals are likely to be localized in a very limited space in the plasma-irradiated liquid. In addition, the plasma jet readily induces DNA strand breaks in synthetic models consisting of tissue fluid, tissue, and cells, without any significant rupture of the phospholipid membrane [33, 34]. Meanwhile, genomic DNA is located inside the nucleus, and thus OH radicals that penetrate through the cell membrane cannot react with the DNA. A possible candidate that increases the intracellular RONS level is the influx of H_2_O_2_ through aquaporins. Hydrogen peroxide, nitrite, and nitrate were generated following He plasma jet irradiation as shown in Fig 2(b). However, Bauer reported that an aquaporin-dependent process may not occur during plasma irradiation [44]. In contrast, Fig 2(c) showed an increase in the intracellular RONS level during plasma irradiation, although the RONS specificity of the probe used here is not good. Hence, OH radicals may be the predominant factor in the DNA damage observed in this study; however further investigation is required to identify the predominant factor(s).

## Conclusion

We demonstrated DNA strand breaks and 8-oxoG generation that were induced by the He plasma jet. Although strand breaks induced by CAP treatment already have been reported, we showed that plasma irradiation that cannot induce strand breaks can induce 8-oxoG generation in DNA. In contrast, plasma irradiation did not result in a notable reduction in cell viability. This could be attributed to up-regulation of the DNA repair process. Cellular responses induced by such weak plasma irradiation have not been well studied; therefore, this type of investigation will contribute to further understanding of these events in plasma medicine.

## Supporting information

S1 Fig Flow cytometric analysis of 8-oxoG production with immunostaining. “He gas 1 min” indicates the control experiment in which the cell suspension was irradiated with He gas flow for 1 minute. Detection of 8-oxoG with immunofluorescence staining was adopted from the method of Ohno et al. [45] and Stanicka et al. [46]. Following APPJ irradiation of A549 cells in one well of a 24-well tissue culture test plate at a concentration of 4.0 × 10^5^ cells/ml in 1 ml of D-PBS (−), the cell suspension was centrifuged at 400 × g for 5 minutes at 4°C, and then the cells were resuspended in 1 ml CELLOTION (Takara Bio) and centrifuged at 400 × g for 5 min at 4°C. The cells were fixed with 4% paraformaldehyde in D-PBS (−) for 1 hour at room temperature and rinsed with CELLOTION twice. To eliminate RNA, cells were treated with 0.1 mg/ml RNase solution for 1 hour at 37°C. After centrifugation at 400 × g for 5 minutes at 4°C, the cells were washed with CELLOTION and then treated with 2N HCl for 20 minutes at room temperature to denature nuclear DNA. Following centrifugation, the cells were treated with 0.1 M sodium borate buffer for 2 minutes at room temperature. Then cells were washed with CELLOTION again and treated with 0.2% bovine serum albumin (BSA), 0.05% saponin in D-PBS (−) for 20 minutes at room temperature. After washing the cells with 3% BSA in D-PBS (−) twice, the cells were stained with an anti-DNA damage antibody labeled with FITC (ab183393, abcam) at 4°C overnight. The following day, the cells were washed with 3% BSA in D-PBS (−) three times, and then analyzed with flow cytometry.
(TIF)
