# Supplementary figures and images for: Strand breaks and chemical modification of intracellular DNA induced by cold atmospheric pressure plasma irradiation
Source: PLoS One. 2020 May 6;15(5):e0232724. doi: 10.1371/journal.pone.0232724 (PMC7202611; doi:10.1371/journal.pone.0232724)

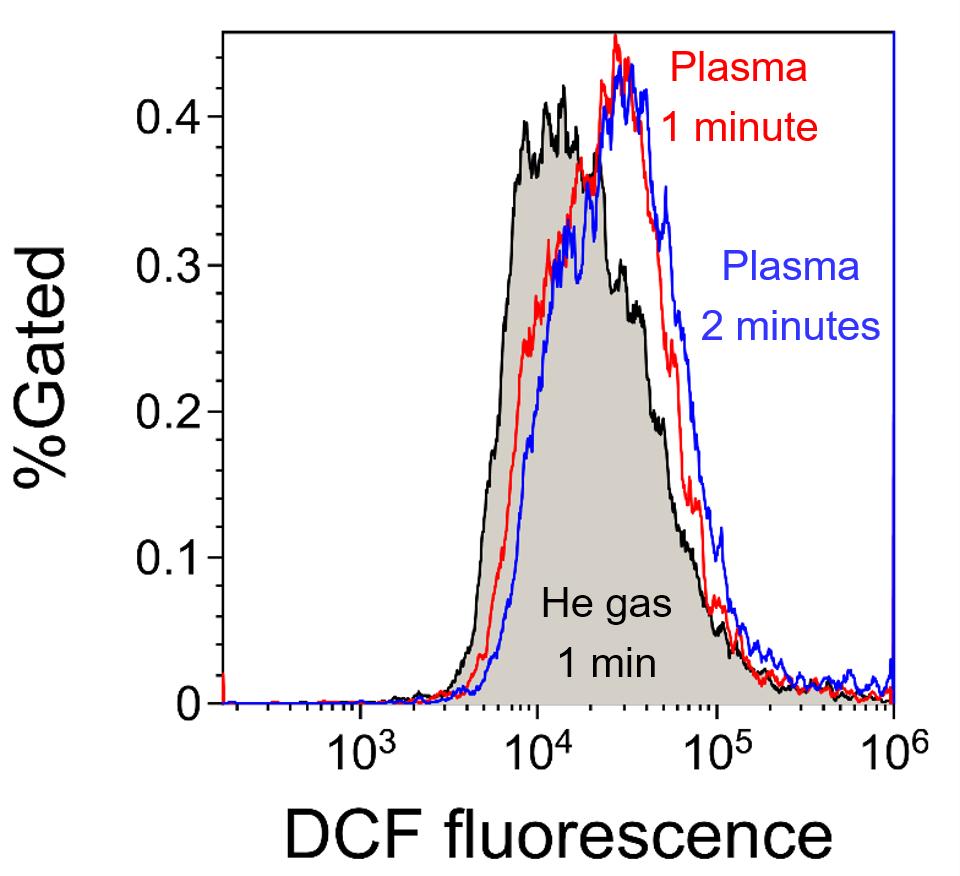

Supplement: S1 Fig — “He gas 1 min” indicates the control experiment in which the cell suspension was irradiated with He gas flow for 1 minute. Detection of 8-oxoG with immunofluorescence staining was adopted from the method of Ohno et al. [45] and Stanicka et al. [46]. Following APPJ irradiation of A549 cells in one well of a 24-well tissue culture test plate at a concentration of 4.0 × 10^5^ cells/ml in 1 ml of D-PBS (−), the cell suspension was centrifuged at 400 × g for 5 minutes at 4°C, and then the cells were resuspended in 1 ml CELLOTION (Takara Bio) and centrifuged at 400 × g for 5 min at 4°C. The cells were fixed with 4% paraformaldehyde in D-PBS (−) for 1 hour at room temperature and rinsed with CELLOTION twice. To eliminate RNA, cells were treated with 0.1 mg/ml RNase solution for 1 hour at 37°C. After centrifugation at 400 × g for 5 minutes at 4°C, the cells were washed with CELLOTION and then treated with 2N HCl for 20 minutes at room temperature to denature nuclear DNA. Following centrifugation, the cells were treated with 0.1 M sodium borate buffer for 2 minutes at room temperature. Then cells were washed with CELLOTION again and treated with 0.2% bovine serum albumin (BSA), 0.05% saponin in D-PBS (−) for 20 minutes at room temperature. After washing the cells with 3% BSA in D-PBS (−) twice, the cells were stained with an anti-DNA damage antibody labeled with FITC (ab183393, abcam) at 4°C overnight. The following day, the cells were washed with 3% BSA in D-PBS (−) three times, and then analyzed with flow cytometry. (TIF) [file pone.0232724.s001.tif]
